# Validity of an online, self-administered Timeline Followback for alcohol use with adolescents

**DOI:** 10.3389/fpsyt.2023.1221487

**Published:** 2023-11-30

**Authors:** Natascha Hareskov Jensen, Lotte Vallentin-Holbech, Genevieve F. Dash, Sarah W. Feldstein Ewing, Kristine Rømer Thomsen

**Affiliations:** ^1^Center for Alcohol and Drug Research, Department of Psychology and Behavioral Sciences, Aarhus University, Aarhus, Denmark; ^2^Department of Psychological Sciences, University of Missouri, Columbia, MO, United States; ^3^Department of Psychology, University of Rhode Island, Kingston, RI, United States

**Keywords:** alcohol use, binge-drinking, Timeline Followback, adolescents, assessment, measurement validation

## Abstract

**Objective:**

The Timeline Followback (TLFB) is a widely used and well-validated interview-based tool for assessing patterns of recent health risk behavior. There is some evidence of the validity of the TLFB as a self-administered online tool for assessing alcohol use, but further research is needed to establish its validity in younger populations and populations outside the United States. Further, it is unknown how self-administered online TLFB formats compare to more timesaving and commonly used single-item alcohol questions. The primary aim of the current study was to validate a new online, self-administered TLFB for alcohol use against the TLFB interview in a sample of European (Danish) adolescents aged 16–18 years (*N* = 30).

**Methods:**

Participants completed a TLFB telephone interview, a self-administered online version of the TLFB, and single-item alcohol questions. Assessments were administered using a within-subject, counter-balanced design. Estimates of number of drinking days, binge-drinking days, maximum drinks consumed on one occasion, total drinks, and drinks per drinking day were compared across metrics.

**Results:**

All correlations between the drinking outcomes assessed via the TLFB interview and the TLFB online were positive, and statistically significant (*r*_s_s = 0.86–0.94, *p* < 0.01). Wilcoxon signed-rank tests showed no significant differences between the TLFB interview and the TLFB online on drinking days, binge drinking days, max drinks, and total drinks. Participants reported drinking significantly more drinks per drinking day on the TLFB online (M = 4.66) compared to on the TLFB interview (M = 4.12; *p* = 0.009).

**Conclusion:**

Overall, the results support the validity of the online, self-administered TLFB in a sample of European (Danish) adolescents.

## 1 Introduction

The Timeline Followback (TLFB) is a widely-used method for assessing recent quantity and frequency of daily health behaviors using an interviewer-based calendar format. It was originally developed by Sobell et al. (1) in the context of measuring alcohol use. Several studies have since found that the interview-based TLFB for alcohol use exhibits sound reliability and validity (2–6) across different populations, including different age groups (7–9) and individuals with and without alcohol use disorders (10, 11).

An advantage of the TLFB is that it generates rich information about a wide range of quantity and frequency constructs, including number of drinking days, average number of drinks per drinking day, number of binge-drinking days, weekly drinking, and maximum units of alcohol consumed on a single occasion. Additionally, it is well-suited to capture drinking patterns and sporadic drinking, particularly in recent (e.g., past 7 days, past 30 days, and past 60 days) windows (12). This might be especially relevant in younger populations, like adolescents, who drink opportunistically and sporadically often depending on availability and seasonality (13).

The interview-based TLFB is a relatively time-consuming method of collecting information about alcohol use compared to single item quantity-frequency questions. Hence, in recent years, the TLFB has been adapted for computer and online delivery (14–17). Online, self-administered versions hold a range of advantages over the interview version, including increasing participant accessibility and convenience. Online surveys also enable researchers to assess youth on technology-based platforms with which they are familiar and regularly engaged. This can strengthen retention rates and reports. Further, online, self-administered formats improve time-efficiency, making data collection in large samples easier, and standardizes delivery, thereby reducing interviewer bias. Lastly, online self-administration increases participants’ sense of anonymity and comfort when disclosing sensitive information (15, 18) and has been found to minimize social desirability bias and underreporting (19), which are key concerns when measuring health risk behavior such as alcohol use (20–22).

Studies examining psychometrics of online TLFB for alcohol use found that it demonstrated good reliability and validity. Namely, several studies show that online versions perform comparably to the TLFB interview format (*r* = 0.83–0.94) (15, 16, 23), and have considerable agreement with measures of alcohol use based on daily diaries (*r* = 0.55–0.88) (17). Furthermore, online, self-administered TLFB for alcohol use exhibit concurrent validity with other related but distinct constructs, like the Alcohol Use Disorder Identification test (AUDIT; *r* = 0.32–0.41) (16, 17, 23), which is a well-established and validated scale measuring symptoms of alcohol use disorder (24).

Existing studies validating online TLFB versions for measuring alcohol have used samples of college students (15, 23, 25), young adults (16), and adult men (17)—i.e., age range from 19 to 24 years. To date, no study has validated an online, self-administered version of the TLFB in a younger sample. This is needed because adolescence is increasingly recognized as a unique neurodevelopmental period, which has significant implications for patterns of alcohol use and related problems (26). Relatedly, there is evidence that adolescents are especially sensitive to mode of survey administration and report higher levels of alcohol use on self-administered questionnaires than on interviewer-administered questionnaires (27). This suggests that self-administered questionnaires may diminish underreporting and thus may more accurately reflect drinking behavior in this age group. Existing validation studies of TLFB online versions were conducted in the United States and there is a need for validation studies from other parts of world, particularly countries with different alcohol cultures. Denmark is interesting in this regard, due to the high rates of heavy drinking among adolescents and young adults [ESPADGroup (28)]. Further, youth alcohol use—even high intensity drinking—is not only widely accepted, but also expected in many social contexts in Danish culture (29).

### 1.1 Present study

While previous studies have supported the validity of online, self-administered modes of the TLFB for measuring alcohol use in older and United States-based samples, there is a need for validating an online, self-administered version of the TLFB in younger samples including adolescents and samples from other parts of the world. To our knowledge, no previous study has examined the validity of an online, self-administered TLFB and the validity of single item questions in the same sample. Thus, the primary aim of the present study was to examine the validity of an online TLFB against a telephone-interview interviewer-administrated TLFB, in a sample of Danish adolescents, on commonly utilized alcohol outcome variables. The secondary aim of the study was to assess the validity of single items against the TLFB telephone interview.

To accomplish the study objectives, we compared the TLFB telephone interview with an online self-administered version of the TLFB and online self-administered single item questions evaluating drinking days, binge drinking days, drinks per drinking day, maximum drinks per occasion, as well as total drinks in the past 30 days. We hypothesized that the online, self-administered TLFB and the TLFB telephone interview, as well as the self-administered single item questions would be strongly correlated. Secondarily, we hypothesized that there would be no significant differences between the TLFB telephone interview and the TLFB online nor the single item questions.

## 2 Materials and methods

### 2.1 Participants

To participate, adolescents had to be aged 15–18 and have engaged in past month alcohol use. Adolescents were recruited through Facebook, word-of-mouth, and adolescents that, in a prior study, had expressed interest in participating in future studies were contacted by email. Participants were offered a gift certificate of approx. $36.50 USD (250 DKK) in return for their participation.

After initial contact, participants received information stating the purpose of the study, explaining what their participation entailed, and clarifying potential risks and benefits. The participant information also detailed the participants’ rights including that participation was voluntary, and that they had the right to terminate their participation at any point in time. Participants were further informed that their data would be treated in concordance with the current Danish legislation. All participants provided informed consent to be part of the study. According to Danish legislation [The Committee Law, the Danish General Data Protection Regulation (GDPR), and the Danish Data Protection Act], individuals aged 16 or above can provide independent informed consent for study participation, provided that the study only collects information by, e.g., interview or survey, that participants are not subjected to any intervention and that study does not involve human biological matter, as is the case in the study in question. As all participants recruited were minimum 16 years old, parental consent was not required for any participants in our final sample. We evaluated whether each participant was adequately mature to provide independent consent via a telephone conversation, which took place prior to conducting the TLFB telephone interview. Specifically, this was done by checking that participants understood the purpose, method, benefits, and potential harms of participating in the study, which is the prerequisite for giving informed consent. Participation was scheduled at the participants’ convenience.

### 2.2 Design and procedure

We used a randomized, counterbalanced within-subject design. Participants were administered all modalities during the same participation event, reporting on their alcohol use in the past 30 days. To eliminate order effects, administration of the online, self-administered TLFB, and the single item questions was counterbalanced. As such, each participant was randomly assigned to complete either the online, self-administered TLFB (Group A) or the single item questions (Group B) first. All participants were administered the TLFB interview as the last part of the study.

To reduce expectancy effects, participants were told that the questionnaire and interview would be about their use of and attitudes toward alcohol. They were not told that they had to complete the same survey in two different modalities, or that the study was about validating different methods of assessing alcohol use.

Further, participants were asked a series of fill/distraction questions about perceived peer approval of substance use, perceived peer substance use, their own attitudes toward peer substance use, their alcohol expectations, and their beliefs about how drinking relates to popularity and friendship (31 items in total). These questions were asked in between administration of all different methods of questioning about alcohol use, to both mask the purpose of the study and reduce the likelihood of participants relying on memory of their answers on the previous administration method, instead of recalling their alcohol use in the past 30 days anew.

### 2.3 Measures

#### 2.3.1 Socio-demographic information

Participants answered demographic questions on age, gender, education, and family socio-economic status. Participants were asked about their perception of their families’ affluence relative to other Danish families (30). This measure was used, instead of, e.g., a measure asking about family income, as adolescents are likely unaware of their parents’ actual income in DKK.

#### 2.3.2 Timeline Followback telephone interview on alcohol use

The Timeline Followback Telephone Interview was completed in line with traditional in-person interview approaches (31).

To mimic the visual aid effect in the in-person TLFB interview, participants were sent a TLFB-calendar by email and a sheet explaining what constitutes a unit of alcohol, including examples of how many units of alcohol common alcoholic beverages correspond to. Interviewees were instructed to use the material as aid throughout the interview. Each interview took 10–15 min to complete.

#### 2.3.3 Online, self-administrated Timeline Followback on alcohol use

For the online, self-administered TLFB, we developed a version that could easily be set up within commonly used and accessible programs for creating online questionnaire-based surveys, and thus be administered as a part of larger surveys (See Appendix 1). The online calendar was constructed in the program SurveyXact, an online tool for generating questionnaire-based surveys (32). Each day of the past 30 days corresponded to one cell in the survey program.

Before filling out the calendar, participants were presented with an explanation of what constitutes a unit of alcohol, including examples of how many units of alcohol common alcoholic beverages correspond to. Participants were given a short, written introductory instruction including to think about special activities or events they attended in the past month (e.g., parties, birthdays, or days hanging out with friends) and to consult their own calendar. The introduction concluded with the instruction to think back on the past 30 days and for each day type in the corresponding cell of the calendar, how many units of alcohol they consumed on a given day, starting with the day before the date of the survey. On days the participants did not consume alcohol, they were instructed to type the number zero.

For both the TLFB interview and the TLFB online, number of drinking days and binge drinking days were calculated as the number of days each participant had reported drinking >0 and > 4 drinks, respectively. Total number of drinks was calculated by adding the number of drinks consumed each day. The maximum number of drinks had on one occasion corresponds to the maximum units of alcohol that the participants had consumed in a single day. The number of drinks per drinking day was calculated as a mean (total number of drinks consumed in the past 30 days divided by number of drinking days).

#### 2.3.4 Single item questions on alcohol use

As part of the online survey, participants were presented with a range of single item questions about their alcohol use in the past 30 days. Frequency of drinking days was measured with: *“Think back on the past 30 days. How many times did you drink alcohol?”* Quantity was measured with: *“Think back on the past 30 days. How many units of alcohol do you usually drink on a day, where you drink alcohol?”* Frequency of binge-drinking days was measured with: *“Think back on the past 30 days. How many times did you drink 5 or more units of alcohol on the same occasion?”* The maximum number of drinks consumed on a single occasion in the past 30 days was measured with: *“What is the largest number of units of alcohol you have been drinking on the same occasion, during the past 30 days?”* The variable total drinks was calculated by multiplying the quantity of alcohol use with the frequency of alcohol use in the past 30 days.

### 2.4 Data analysis

This study included a sample size of *n* = 30 participants, which is considered sufficient for the Central Limit Theorem to hold and to provide a reasonable level of power for continuous data.

Frequencies of demographic variables were calculated. Fisher’s exact tests and independent *t*-tests were conducted to check for potential differences on demographic variables and alcohol use variables (measured by the TLFB interview) between Group A and Group B.

In line with the analytic approach taken in previous studies aiming to establish the validity of online versions of the TLFB, we examined correlations and differences between modes of assessment to test the psychometric properties of the TLFB online (15, 16, 23). Thus, Spearman’s rank correlations were used as the primary analysis to assess the validity of the TLFB online and the single items compared to the TLFB interview and secondarily Wilcoxon signed rank tests were conducted to examine potential within-subject differences on comparable variables between the TLFB online and the TLFB interview as well as between the single items and the TLFB interview. Correlations and differences between the TLFB interview vs. theTLFB online and the single item questions on the variables number of drinking days, binge-drinking days, maximum drinks consumed on one occasion, total drinks, and drinks per drinking day in the past 30 days were examined.

## 3 Results

### 3.1 Characteristics of study sample

Participants (*N* = 30) were 16–18 years old, with a mean age of 17.47 (SD = 0.68). The majority were female (66.7%) and reported that their family was above average affluent compared to other Danish families (60.0%; See Table 1).

**Table 1:** Characteristics of study sample.

	Group A (*N* = 14)	Group B (*N* = 16)	Total (*N* = 30)	*p* value
Demographics				
Age [Mean (SD)]	17.43 (0.85)	17.50 (0.52)	17.47 (0.68)	0.788^b^
Gender [*N* (%)]				0.709^a^
Female	10 (71.4%)	10 (62.5%)	20 (66.7%)	
Male	4 (28.6%)	6 (37.5%)	10 (33.3%)	
(Relative) family affluence [*N* (%)]				0.744^a^
Much better off	2 (14.3%)	5 (31.3%)	7 (23.3%)	
Better off	5 (35.7%)	6 (37.5%)	11 (36.7%)	
About the same	6 (42.9%)	4 (25%)	10 (33.3%)	
Less well off	1 (7.1%)	1 (6.3%)	2 (6.7%)	
TLFB interview [Mean (SD)]				
Drinking days, past 30 days	6.50 (2.21)	6.50 (3.76)		1.000^b^
Binge drinking days, past 30 days	1.93 (1.86)	2.00 (2.42)		0.929^b^
Max drinks/occ. past 30 days	8.39 (4.63)	9.75 (6.87)		0.537^b^
Drinks per drinking day, past 30 days	3.99 (2.15)	4.23 (3.05)		0.806^b^
Total drinks, past 30 days	25.82 (17.84)	26.69 (22.99)		0.910^b^
Single items [Mean (SD)]				
Drinking days, past 30 days	6.50 (3.03)	5.38 (3.89)		0.390^b^
Binge drinking days, past 30 days	2.50 (2.28)	2.25 (2.41)		0.773^b^
Max drinks/occ. past 30 days	8.43 (5.16)	10.40 (7.18)		0.406^b^
Drinks per drinking day, past 30 days	4.57 (3.37)	7.64 (6.42)		0.125^b^
Total drinks, past 30 days	29.79 (24.21)	34.43 (29.34)		0.652^b^

^a^Fisher’s exact test. ^b^Independent *t*-test.

As seen in Table 1, Fisher’s exact tests showed no significant associations between group allocation and either gender or family affluence. Independent sample *t*-tests further showed no significant differences in age or on any alcohol use variables as measured by either the TLFB interview or the single item questions between participants randomized into being administrated the TLFB online (Group A) and the single item questions (Group B) as the first method of questioning (*p* > 0.05). Thus, no order effects were detected.

### 3.2 Correlations and differences

Correlations between the TLFB interview and the online format on all variables were positive and statistically significant (*p* < 0.001). Further, all correlations between the TLFB interview and the single item questions were also positive and statistically significant (*p* < 0.001; See Table 2).

**Table 2:** Comparison of TLFB telephone interview, online self-administered TLFB, and self-administered single item questions on alcohol use during the past 30 days.

	Mean (Range)			Spearman’s Rho correlations		*p* value based on Wilcoxon signed rank test	
Mode Variable	TLFB interview	TLFB online	Single items	TLFB interview vs. online	TLFB interview vs. single items	TLFB interview vs. online	TLFB interview vs. single items
Number of drinking days	6.50 (13.00)	6.13 (16.00)	5.90 (14.00)	0.94^*^	0.89^*^	0.236	0.073
Number of binge drinking days	1.97 (9.00)	2.17 (10.00)	2.37 (8.00)	0.86^*^	0.83^*^	0.144	0.106
Maximum number of drinks on one occasion	9.12 (23.00)	9.13 (28.00)	9.45 (28.00)	0.94^*^	0.94^*^	0.907	0.351
Number of drinks per drinking day	4.12 (9.75)	4.66 (10.71)	6.11 (24.00)	0.93^*^	0.71^*^	0.009^*^	0.072
Total number of drinks consumed	26.28 (82.00)	26.33 (88.00)	32.11 (97.00)	0.94^*^	0.81^*^	0.413	0.309

^*^p < 0.01.

We further found no significant within-subject differences between the TLFB online and interview variables for drinking days, binge drinking days, maximum drinks, and total drinks. The only significant difference found was on drinks per drinking day, where participants reported drinking significantly more drinks on the TLFB online compared to on the TLFB interview format. No significant differences were found between the TLFB interview and the single item questions on any variables (see Table 2).

It is worth noting that closer examinations of descriptive statistics revealed that the single item quantity question measuring drinks per drinking day exhibited much larger variability than the TLFB interview and TLFB online drinks per drinking day variables. For example, the range of the single item quantity variable was more than two times as large as the range of either of the TLFB drinks per drinking day variables. This seemed to mainly be due to a share of participants tending to report a discrepantly large number of drinks per drinking day on the single item.

## 4 Discussion

### 4.1 Validity of the TLFB online

These preliminary data support the validity of the online, self-administered TLFB against the TLFB telephone interview. All correlations between variables derived from the TLFB interview and the TLFB online were positive and significant. This reflects findings from earlier studies, comparing online versions and interview versions of the TLFB among older samples, which also found positive and significant correlations: *r* = 0.85–0.94 for total drinks, *r* = 0.89–0.91 for drinking days, *r* = 0.78–0.88 for drinks per drinking days, *r* = 0.88 for maximum drinks and *r* = 0.83 for binge drinking days (15, 16, 23).

On the variables drinking days, binge drinking days, maximum drinks on one occasion, and total drinks, no significant differences were found between the TLFB online and TLFB interview. However, one significant difference was found between the two modes on the variable drinks per drinking day. Thus, on the TLFB online, participants reported drinking significantly more drinks per drinking day than on the TLFB interview. One explanation for this pattern is that it may simply be harder for participants to accurately recall their alcohol use on the TLFB online than on the TLFB interview due to the absence of interviewer assistance in recalling use, participants misunderstanding written instructions, and/or confusion regarding the survey set-up. However, as others have noted, online, self-administered surveys promote participants’ sense of anonymity and comfort (15, 18), and thus reduce social desirability bias and underreporting (19), which is particularly important in this adolescent age group. Thus, the significant difference found on the variable drinks per drinking day between the two modes of the TLFB might alternatively be due to participants being less inclined to underreport the number of drinks consumed on the TLFB online, due to this measure being perceived as more confidential.

Existing online versions of the TLFB typically involve rather elaborate, interactive set-ups and instructions [e.g., (14, 17)]. For this study, we developed a TLFB format with a simple set-up and simple instructions that is easy and quick for participants to acquaint themselves with (See Appendix 1). This streamlined format can also be easily and seamlessly incorporated into larger surveys, using standard survey generating tools/software, which might be advantageous, as the use of such software have become increasingly popular in recent years (33).

### 4.2 Validity of single item questions

Correlations between the TLFB interview and the single-item questions on all measured variables were also positive and significant. No significant differences were found between the TLFB interview and the single item questions on any of the variables. While the drinks per drinking day variable was calculated in the same way for both the TLFB online and the TLFB interview (i.e., as the mean number of drinks), quantity single item questions asking about how many units of alcohol the participant “typically “or “usually” drink might not be interpreted by participants as asking for a mean, and might instead elicit answers corresponding to a median or mode of drinks consumed on drinking days (34).

The single-item drinks per drinking day variable exhibited larger variability than the TLFB interview and the TLFB online drinks per drinking day variables, and more participants were likely to report a discrepantly large number of drinks per drinking day on the single item question than on the TLFB interview and online formats. Moreover, inspection of data further revealed that both in total and for the majority of individual participants, the absolute differences between the TLFB interview and the TLFB online drinks per drinking day variables were smaller than the absolute differences between the TLFB interview and the single item quantity question variable. These tendencies could be due to a proportion of participants misinterpreting the ambiguously phrased quantity question as asking for something other than a mean. All in all, this could point to a weakness in the single item quantity question that might not have been caught by the Wilcoxon signed-rank test, which is based on ranking score differences, and thereby is insensitive to absolute magnitudes of differences and outliers.

### 4.3 Strengths, limitations, and future directions

This study should be interpreted with these limitations in mind; First and foremost, this was a preliminary study with a small sample size. In turn, next steps will include evaluation of our online measure with a larger, better-powered sample to build on and extend these findings. Our work continues to examine how to use a neurodevelopmental approach to model, and measure alcohol use disorders and hazardous drinking in the adolescent age group, specifically, and these data are a crucial step in this line of study. Second, other similar studies administered the two modalities of the TLFB several days apart (15, 16, 23). Due to practical reasons, concern about drop-out and consideration for participants’ time, both modes of administration of the TLFB and the single-item questions were completed in the same sitting in the current study. This could theoretically have increased the risk that participants relied on their memory of their recent answers on previous methods of questioning, when completing subsequent methods, which may have led to an overestimation of the validity of the TLFB online and the single item questions. In this regard, it is a limitation, that this study only counterbalanced administration of the TLFB online and the single item questions, and thus did not randomize order of completion of all three measures examined, to be able to check for possible order effects due to all participants completing the TLFB interview as the last measure. Lastly, the analytical strategy utilized in this study was chosen to facilitate comparisons with prior studies examining the psychometric properties of online version of the TLFB. However, it is worth noting that the statistical approach, using Wilcoxon signed-rank tests, is framed from the null hypotheses that there would be no significant differences between modes of administration. Thus, our findings should be interpreted accordingly—as indicating that there were no significant differences between measures—rather than be interpreted as an indication of similarity in performance across the measures examined in this study.

At the same time, this study has several strengths. First of all, this study is, to our knowledge, the first to examine the validity of both single-item questions and an online version of the TLFB in the same sample, making it possible to compare the performance of the two. Second, this study counterbalanced administration of the TLFB online and single item questions to eliminate order effects on estimates of the validity of these measures. Third, the use of fill/distraction questions may have served to both reduce expectancy and carry-over effects, specifically the likelihood of participants relying on the memory of their answers on the previous administration method, instead of recalling their alcohol use in the past 30 days anew. This study also provides novel insight into the performance of these measures in a European (Danish) sample of adolescents.

In sum, the study lends preliminary support to the validity of a novel, online and self-administered TLFB in a sample of European (Danish) adolescents. Future studies should replicate this finding in a larger sample.

## Data availability statement

The datasets presented in this article are not readily available because the Danish Data Protection Regulation, Data Protection Act, and Health Act restrict sharing of data, and we would have to confer with the legal department at the University of Aarhus, to make sure, that it is legitimate for us to share the data. Requests to access the datasets should be directed to nhj@psy.au.dk.

## Ethics statement

Requirement of ethical approval was waived by the Central Denmark Region Committees on Health Research Ethics. In accordance with the Consolidation Act on Research Ethics Review of Health Research Projects, Consolidation Act number 1083 of 15 September 2017 section 14 (1), only health research studies have to be notified to the Committees. The Central Denmark Region Committees on Health Research Ethics did not consider this study to be a health research study [section 2 (1)], and, therefore, from the Committees was not required. The study was conducted in accordance with the local legislation and institutional requirements. According to the Danish General Data Protection Regulation (GDPR) and the Data Protection Act, written informed consent for participation was not required from the participants’ legal guardians/next of kin and the participants were able to independently give informed consent to participate in the study, because a) the study only involved the collection of data by interview and survey, b) study participants were not subjected to any intervention and c) the study did not involve human biological matter. The information provided prior to the participants consenting was phrased in a clear and easily understandable language, ensuring participant apprehension. Only individuals, who were evaluated to be adequately mature to consent by themselves, were included in the study. Adequate maturity was assessed by talking to participants via telephone prior to conducting the TLFB-T interview.

## Author contributions

NH wrote the first draft of the manuscript, collected data, and performed the statistical analyses. KR led the conception and design of the study with contribution from SF, LV-H, and NH. NH, LV-H, GD, SF, and KR contributed to the statistical analyses and to the manuscript, critically reviewed its content, and approved the submitted version. All authors contributed to the article and approved the submitted version.
