# Supplementary material for: Validity of an online, self-administered Timeline Followback for alcohol use with adolescents
Source: Front Psychiatry. 2023 Nov 30;14:1221487. doi: 10.3389/fpsyt.2023.1221487 (PMC10720705; doi:10.3389/fpsyt.2023.1221487)
Supplement: Supplementary file 1 [file Image_1.pdf]

Your alcohol use the past 30 days.

To gain an overview of you alcohol use the past month, we are going to ask you to write down how much you had to drink each day during the past 30 days.

To help you remember, we have made a table resembling a calendar. Begin by thinking of any special activities you participated in or things you did in the past month, e.g., days where you went to a party, someone's birthday or where you got together with friends. If possible, consult your own calendar.

We realize that it is hard for anyone to recall things with I00-percent accuracy, but please try to be as accurate as possible.

Write in the calendar/tabel on the next page how many units of alcohol you drank on each day.

Think back on the past 30 days and for each day, write the number of units of alcohol, you drank on that day in the calendar. If you did not drink any units on a given day, please write 0. Begin with yesterday, the 25th of October (at the bottom of the calendar).

	Monday	Tuesday	Wednesday	Thursday	Friday	Saturday	Sunday
4 weeks ago	Sept. 26	Sept.27	Sept. 28	Sept. 29	Sept. 30	Oct. 1	Oct. 2
3 weeks ago	Oct. 3	Oct. 4	Oct. 5	Oct. 6	Oct. 7	Oct. 8	Oct. 9
2 weeks ago	Oct. 10	Oct. 11	Oct. 12	Oct. 13	Oct. 14	Oct. 15	Oct. 16
Last week	Oct. 17	Oct. 18	Oct. 19	Oct. 20	Oct. 21	Oct. 22	Oct. 23
This week	Oct. 24	Oct. 25					
